# Association between S100A12 and risk of peripheral arterial disease in patients with dyslipidemia: a cross-sectional study

**DOI:** 10.1186/s12872-025-04752-2

**Published:** 2025-04-24

**Authors:** Wenyu Cai, Yilin He, Guohua Li, Dengqing Zhang, Zimin Chen, Shijia Jin, Yifan Zhang, Zhong Chen

**Affiliations:** 1 Department of Cardiology, Shanghai Sixth People’s Hospital Fujian, Jinjiang, Fujian 362200 P.R. China; 2 Department of Cardiology, Shanghai Sixth People’s Hospital, Shanghai Jiao Tong University School of Medicine, Shanghai, 200233 P.R. China; 3 Department of Clinical Laboratory, Shanghai Sixth People’s Hospital Fujian, Jinjiang, Fujian 362200 P.R. China; 4 Department of Critical Care Unit, Shanghai Sixth People’s Hospital, Shanghai Jiao Tong University School of Medicine, Shanghai, 200233 P.R. China

**Keywords:** Peripheral arterial disease, S100A12, Dyslipidemia, Low-density lipoprotein cholesterol

## Abstract

**Objective:**

S100A12 acts as a pro-inflammatory agent in vivo, with a close relationship with plaque formation in patients with acute coronary syndrome (ACS), end-stage renal disease, and diabetes. Peripheral arterial disease (PAD) can lead to mobility difficulties and ultimately disability and amputation. The association between S100A12 and risk of peripheral arterial disease remains unclear. This study aims to investigate the association between S100A12 and the risk of PAD in patients with dyslipidemia.

**Methods:**

From March 2023 to June 2024, 478 patients were included in this cross-sectional study. They were divided into PAD group (*n* = 105) and control group (*n* = 373) according to the presence or absence of PAD (The diagnosis of PAD is a combination of the patient’s clinical symptoms, imaging evidence and ankle-brachial index). Plasma S100A12 was detected by available kit. General information, disease history, smoking history, and laboratory indicators were collected from both groups. The relationship between S100A12 and the risk of PAD was analyzed using statistical methods.

**Results:**

Levels of S100A12 were significantly higher in the PAD group of dyslipidemia [0.22 (0.13,1.49) ng/cL vs. 0.13 (0.10,0.18)ng/cL, *p* value < 0.001]. Univariate and multivariate logistic regression analyses suggested that the risk of PAD was significantly higher with increasing levels of S100A12 [Odd ratio (OR) (95%CI) = 2.264 (1.681, 3.047), *p* value < 0.05]. In addition, lower high-density lipoprotein cholesterol (HDL-C) level and diabetes mellitus (DM) were independent risk factors for PAD [OR (95%CI) = 0.388 (0.186,0.809), *p* value = 0.012; OR = 2.375 (1.527,3.695), *p* value < 0.001]. Subgroup analysis suggested that S100A12 was significantly and positively associated with the risk of PAD in all subgroups, regardless of whether HDL-C levels < 1.03 mmol/L, age > 60 years, and presence of diabetes or hypertension. Restricted cubic spline (RCS) curves suggested that the correlation between S100A12 and the risk of PAD was nonlinear (*p*-non-linear value < 0.05). The RCS curves showed that the positive correlation between S100A12 and the risk of PAD was stronger when the S100A12 level was less than 1.00ng/cL.

**Conclusion:**

In conclusion, elevated S100A12 level is an independent risk factor for PAD in patients with dyslipidemia. In different subgroups, S100A12 was significantly and positively associated with the risk of PAD after adjusting for different factors. There is a non-linear relationship between S100A12 and the risk of PAD, with a stronger positive correlation at S100A12 levels below 1.00ng/cL. These findings implied that S100A12 is a potential biomarker for identifying patients with dyslipidemia who are at high risk of developing PAD. They also implied that S100A12 levels can be routinely monitored in dyslipidemic populations for the early detection of PAD and to guide the management of PAD. Finally, the results of this study emphasize that inflammation in dyslipidemia patients plays an important role in the development of PAD, suggesting that lipid control and immunomodulation may be effective in the prevention of PAD.

**Clinical trial number:**

MR-35-24-038431.

**Supplementary Information:**

The online version contains supplementary material available at 10.1186/s12872-025-04752-2.

## Introduction

Peripheral arterial disease (PAD) is a disease caused by atherosclerosis, leading to narrowing or even blockage of the peripheral arteries, and ultimately leads to insufficient blood supply to tissues and organs [1]. PAD has shown a marked increase in global prevalence over recent decades [2]. It is estimated that at least 113 million people worldwide, and possibly as many as 236 million, suffer from PAD, although there is significant variation in the estimates of its prevalence [3]. PAD is associated with significantly increased risks of cardiovascular mortality and morbidity [2, 4]. However, current evidence suggests that PAD remains substantially underdiagnosed and undertreated compared to other cardiovascular diseases [2].

To stratify the risk for patients, studies analyzed the risk factors for mortality in PAD and found that age, hypertension, diabetes, smoking, and dyslipidemia are associated with an increased 10-year mortality rate in PAD patients [5, 6]. Lipid profiles, particularly LDL-C (low-density lipoprotein cholesterol), play a critical role in PAD. Many studies have shown that lipid levels, especially LDL-C, are important risk factors for PAD [7–9]. LDL-C on the one hand accumulates in the vascular wall and allows the formation of atherosclerotic plaques, and on the other hand promotes inflammation, which contributes to the progression of plaques [10].

S100A12, a calcium-binding protein secreted by neutrophils and macrophages, acts as a pro-inflammatory agent in vivo by binding to the receptor for advanced glycation end products (RAGE) and stimulating the transduction of a series of intracellular signaling pathways [11]. The binding of RAGE and its ligands is thought to play an important role in inflammation during atherosclerosis [12, 13]. The binding of S100A12 to the RAGE activates intracellular signaling pathways such as mitogen-activated protein kinase (MAPK) and nuclear factor-κB(NF-κB), thereby inducing the production of inflammatory cytokines [such as tumor necrosis factor-α(TNF-α), Interleukin-1β(IL-1β)] and adhesion molecules [such as intercellular adhesion molecule-1(ICAM-1), vascular cell adhesion molecule-1(VCAM-1)] [14]. These cytokines and adhesion molecules work together to recruit the inflammatory cells such as macrophages, neutrophils to infiltrate the vessel wall, exacerbating vascular inflammation, which leading to PAD. It has also been found that binding of S100A12 to RAGE activates Rac1, which triggers NADPH oxidase1 (Nox1)-dependent formation of reactive oxygen species (ROS) and promotes atherosclerosis. It has also been shown that S100A12 binds to CD36, which promotes oxLDL uptake and the formation of foam cells, exacerbating lipid core formation and plaque development [15]. It has been shown that there is a significant correlation between S100A12 and the risk of atherosclerosis in patients with diabetes mellitus (DM) and end-stage renal disease [16, 17]. It has also been shown that S100A12 levels are significantly elevated in patients with coronary artery disease and are a potential predictor of CAD [18, 19].

The correlation between S100A12 and PAD risk in patients with dyslipidemia is still unclear. It is reasonable to hypothesize that the inflammatory response mediated by S100A12 promotes the formation and progression of atherosclerosis in people with dyslipidemia, thereby increases the risk of PAD. The present study was conducted to validate the above hypothesis.

## Methods

### Study population

This cross-sectional study was conducted at the Department of Cardiology, Shanghai Sixth People’s Hospital Fujian from March 2023 to June 2024. A total of 610 subjects were consecutively enrolled in the study. 518 patients over the age of 18 with dyslipidemia were included in the study. The criteria for dyslipidemia were referred to the “Chinese guidelines for lipid management (2023)” [20]. Patients were classified as having dyslipidemia if their baseline lipid levels failed to meet the lipid control targets corresponding to their risk stratification category (Tables 1 and 2).

**Fig. 1:**
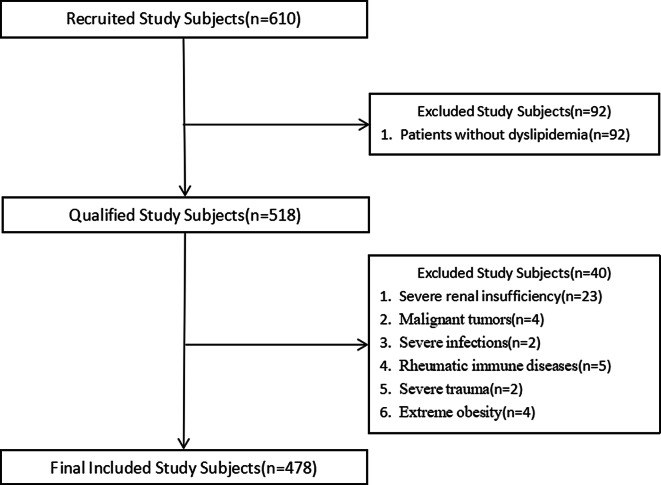
Flowchart

The exclusion criteria are as follows: (1) severe renal insufficiency[eGFR ≤ 30 ml/(min*1.73 m²)]; (2) patients with malignant tumors; (3) patients with severe infections[sequential organ failure assessment (SOFA) > 2]; (4) patients with rheumatic immune diseases; (5) patients with severe trauma[abbreviated injury scale (AIS) > 3]; (6) patients with extreme obesity(BMI > 40 kg/m²). Samples with undiagnosed dyslipidemia and PAD due to missing data were excluded. Finally, 478 patients were included in the study (Fig. 1). Patients enrolled in the study would be collected for age, gender, height, weight, history of diseases, and smoking history. In addition, fasting venous blood would be drawn to obtain white blood cell count, creatinine, various lipid profiles, and glycosylated hemoglobin (HA1c) levels. Lipid profiles were tested by the kits purchased from Sichuan Mack Biological Co and LABOSPECT 008AS automatic biochemistry analyser (Hitachi, Japan) was used. WBC count was measured by XN-550 automatic modular blood and fluid analyser (Sysmex, Japan). HbA1c was measured by D100 Automatic Glycated Haemoglobin Analyser (Borel, USA) and the accompanying reagents. The calculating formulas for body mass index (BMI) and estimated glomerular filtration rate (eGFR) are shown below:

BMI (kg/m^2^)= (weight)/ (height^2^)

eGFR (Male)[ml/ (min*1.73m^2^)] = 186× (creatinine)^−1.154^× (age)^−0.203^

eGFR (Female)[ml/ (min*1.73m^2^)] = 186× (creatinine)^−1.154^× (age)^−0.203^ × 0.742

This study is in accordance with the 2013 revision of the Declaration of Helsinki. This study was approved by the Ethics Committee of Shanghai Sixth People’s Hospital Fujian (jjsyyyxll-2022030).The Clinical Trial Number is MR-35-24-038431.

### Diagnosis of peripheral arterial disease

PAD was diagnosed in patients who met all of the following criteria:

1. Clinical symptoms such as rest pain, claudication symptoms, and ischaemic ulcers.
2. With imaging evidence such as arterial ultrasound doppler.
3. Ankle-brachial blood pressure index (ABI) ≤ 0.80 in patients without a perior history of limb revascularization and ≤ 0.85 in patients with a perior history of limb revascularization [21].

### Quantitative detection of S100A12

Fasting venous blood was drawn from the patients. Ethylenediaminetetraacetic acid (EDTA) anticoagulated samples were immediately centrifuged at 2000 g for 10 min, and plasma was placed in a refrigerator at -80℃ for assay. The concentration of S100A8/A9 and S100A12 were measured by ELISA method. And the kits were purchased from Shanghai Yansheng Biotechnology Industrial Co, LTD,. The analysisers were unaware of the subject’s diagnosis.

### Statistical methods

The Kolmogorov-Smirnov test was used to check the normality of the distribution. All continuous variables included in this study did not conform to normal distribution after the normal distribution test. Therefore the analysis of the difference of continuous variables between the PAD and control groups was performed using the Kruskal-Wallis H test. Continuous variables were presented in quartiles. The study of the difference in categorical variables between the two groups was performed using the chi-square test. Categorical variables were presented as percentages. Available case analysis is used to handle missing data. Logistic regression was used for univariate and multivariate analysis of risk factors for PAD. The association between S100A12 and the risk of PAD was analyzed in subgroups based on whether patients were smokers, had DM or hypertension (HTN), ≥ 60 years, and had high-density lipoprotein cholesterol (HDL-C) ≥ 1.03 mmol/L. Restricted cubic spline (RCS) curves were used to explore the nonlinear relationship between S100A12 and the risk of PAD. Software packages R and SPSS29.0 were used to analyze the data. All statistical tests were two-sided, and p-values less than 0.05 were considered statistically significant differences.

## Result

### Baseline characteristics of the study subjects

As shown in Table 1, a total of 478 patients were enrolled in the study, including 105 in the PAD group and 373 in the control group. The patients in the PAD group were older compared to the control group, while the level of HDL-C was significantly lower, the level of HA1c and the percentage of hypertensive and diabetic patients was significantly higher (all *p* value < 0.05). The level of S100A12 was higher in the PAD patients compared to the control group [0.22 (0.13,1.49) vs. 0.13 (0.10,0.18), *p* value < 0.001].

**Table 1:** Baseline characteristics of the study subjects

	PAD group (*N*=105)	Controls (*N*=373)	*p* value
General information			
Gender			
Female (n/%)	23 (21.9)	106 (28.4)	0.184
Male (n/%)	82 (78.1)	367 (71.6)	
Age (years)	65 (57,70)	62 (53,70)	0.045
BMI (kg/m^2^)	24.2 (22.5,26.7)	24.3 (22.0,27.2)	0.893
History of diseases and smoking			
Coronary artery disease (n/%)	33 (31.4)	139 (37.3)	0.271
Stroke (n/%)	11 (10.5)	28 (7.5)	0.326
HTN (n/%)	71 (67.6)	216 (57.9)	0.029
DM (n/%)	53 (50.5)	112 (30.0)	<0.001
Smoking (n/%)	53 (50.5)	163 (43.7)	0.218
Laboratory indicators			
WBC (*10^9^)	7.27 (5.83,8.91)	7.41 (5.86,9.13)	0.825
Creatinine (μmol/L)	80.50 (65.96,93.45)	75.35 (63.53,89.52)	0.111
eGFR[ml/ (min*1.73m^2^)]	87.50 (73.25,106.50)	93.50 (76.00,107.00)	0.224
TC (mmol/L)	4.77 (3.86,6.10)	4.82 (4.06,5.72)	0.736
TG (mmol/L)	1.43 (1.02,1.90)	1.48 (1.07,2.11)	0.485
LDL-C (mmol/L)	2.78 (1.98,3.66)	2.96 (2.32,3.71)	0.131
HDL-C (mmol/L)	1.11 (0.94,1.32)	1.16 (0.98,1.39)	0.047
HA1c (%)	7.90 (7.20,9.00)	7.50 (6.20,8.70)	0.030
S100A8/A9 (ng/cL)	0.10 (0.06,0.94)	0.09 (0.06,0.14)	0.069
S100A12 (ng/cL)	0.22 (0.13,1.49)	0.13 (0.10,0.18)	<0.001

### Univariate logistic regression analyses for the risk predictors of PAD

**Fig. 2:**
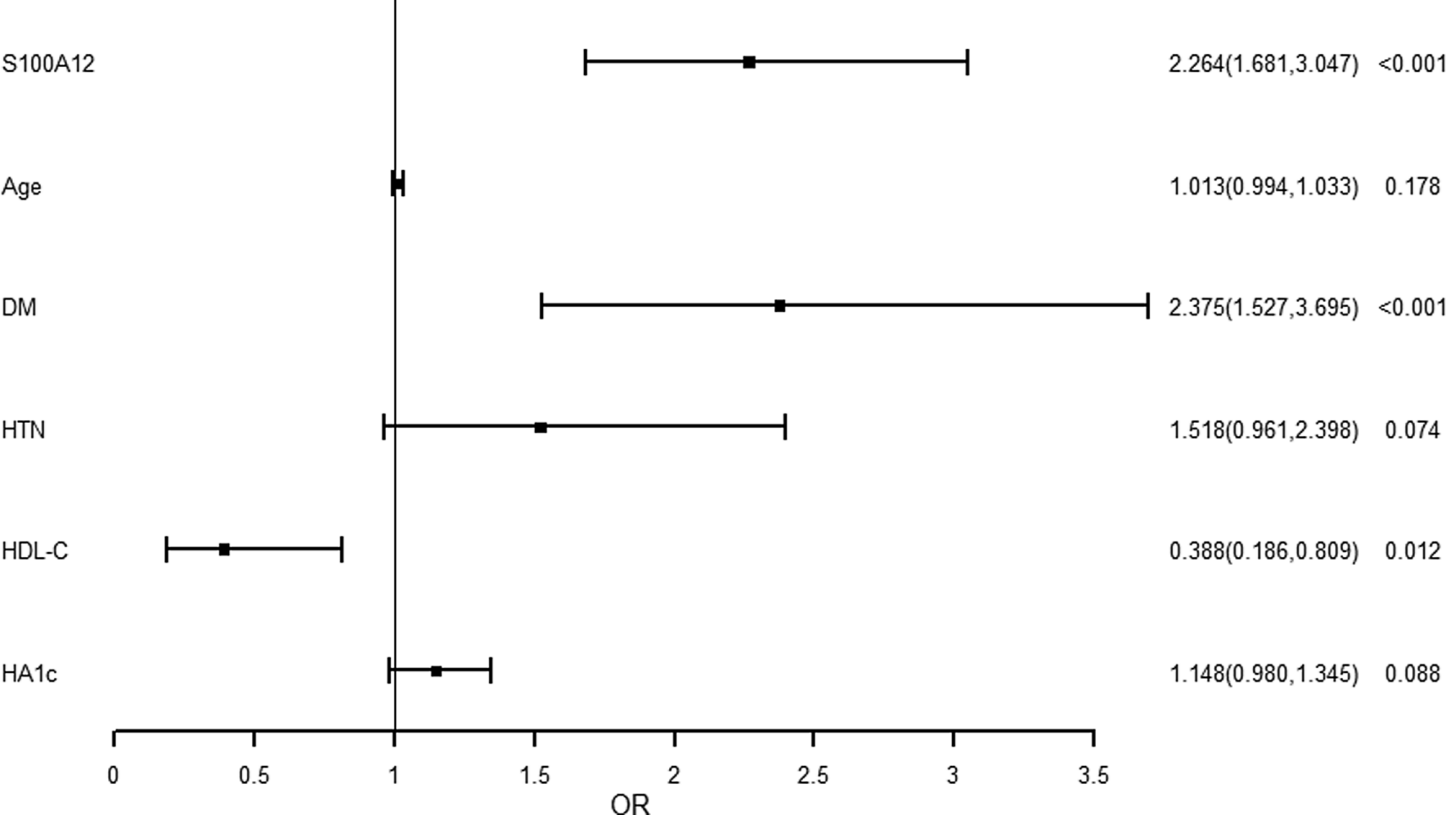
Univariate logistic regression analysis of risk factors for PAD

As shown in Fig. 2, univariate logistic regression analyses revealed that diabetes and plasma S100A12 levels were promotive factors for PAD, whereas HDL-C levels were protective factors for PAD.

### Subgroup analyses of the relationship between S100A12 levels and PAD risk

**Fig. 3:**
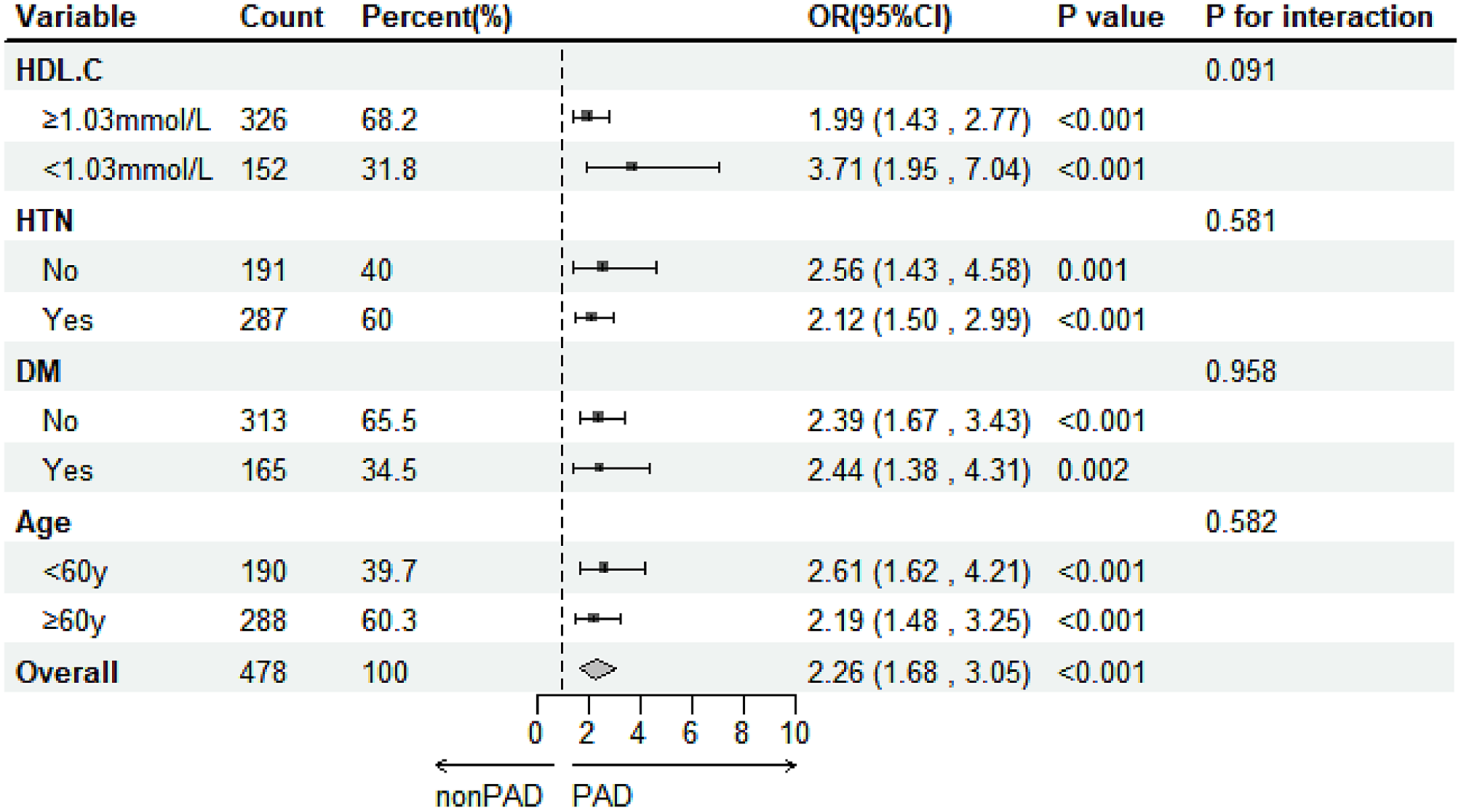
Subgroup analyses of the relationship between S100A12 levels and PAD risk without adjusting for different factors

As shown in Fig. 3, subgroup analyses of patients by HDL-C level, age, HTN and DM showed a significant association between S100A12 and the risk of PAD in all subgroups (all *p* value < 0.05).

### Model of the relationship between S100A12 and PAD after adjusting for different risk predictors

As shown in Table 2, S100A12 levels were positively correlated with the risk of PAD significantly in different models adjusting for different factors. The participants were then grouped by quartiles of S100A12 levels. In the different models, the Q4 group significantly increased the risk of PAD compared to the Q1 group, which indicates that elevated levels of S100A12 increase the risk of PAD.

**Table 2:** Model of the relationship between S100A12 and PAD after adjusting for different risk predictors

OR (95%CI)			
Participants	Crude	Model1	Model2
All participants	2.264 (1.681,3.047)^***^	2.439 (1.795,3.316)^***^	2.469 (1.810,3.368)^***^
S100A12 (ng/cL)			
Q1 (≤ 0.049)	Ref	Ref	Ref
Q2 (0.049–0.066)	2.198 (0.982,4.919)	2.558 (1.123,5.825)^*^	2.583 (1.129,5.911)^*^
Q3 (0.066–0.115)	2.984 (1.363,6.531)^**^	3.433 (1.540,7.652)^**^	3.554 (1.587,7.957)^**^
Q4 (> 0.115)	8.162 (3.882,17.158)^***^	9.683 (4.494,20.862)^***^	10.105 (4.650,21.957)^***^
P for trend	< 0.001	< 0.001	< 0.001

Note: Crude: Unadjusted;

Model 1: Adjusted for DM, and HDL-C;

Model 2: Adjusted for age, DM, HTN, and HDL-C

*:*p* value < 0.05; **: *p* value < 0.01, ***: *p* value < 0.001

### Subgroup analysis of each model after adjusting for different factors

As shown in Table 3, S100A12 levels were positively associated with the risk of PAD in all subgroups of the different models. Subgroup analyses also suggested that the correlation between S100A12 and the risk of PAD was stronger in younger (< 60y) or lower HDL-C (< 1.03 mmol/L) patients.

**Table 3:** Subgroup analysis of each model after adjusting for different factors

OR (95%CI)			
Participants	Crude	Model1	Model2
HDL-C			
<1.03mmol/L	3.706 (1.951,7.041)^***^	4.263 (2.163,8.404)^***^	4.849 (2.370,9.921)^***^
≥1.03mmol/L	1.989 (1.428,2.770)^***^	2.059 (1.462,2.899)^***^	2.054 (1.458,2.894)^***^
Age			
≥60years	2.193 (1.478,3.255)^***^	2.403 (1.594,3.622)^***^	2.445 (1.608,3.716)^***^
<60years	2.611 (1.617,4.214)^***^	2.920 (1.743,4.893)^***^	2.916 (1.730,4.915)^***^
Smoking			
Yes	2.897 (1.851,4.535)^***^	2.992 (1.905,4.700)^***^	3.176 (1.972,5.117)^***^
No	1.799 (1.216,2.662)^**^	2.143 (1.389,3.305)^**^	2.134 (1.382,3.297)^**^

Note: Crude: Unadjusted;

Model 1: Adjusted for DM, and HDL-C;

Model 2: Adjusted for age, DM, HTN, and HDL-C

*:*p* value < 0.05; **: *p* value < 0.01, ***: *p* value < 0.001

### Analyzing the non-linear relationship between S100A12 and PAD risk using RCS curves

**Fig. 4:**
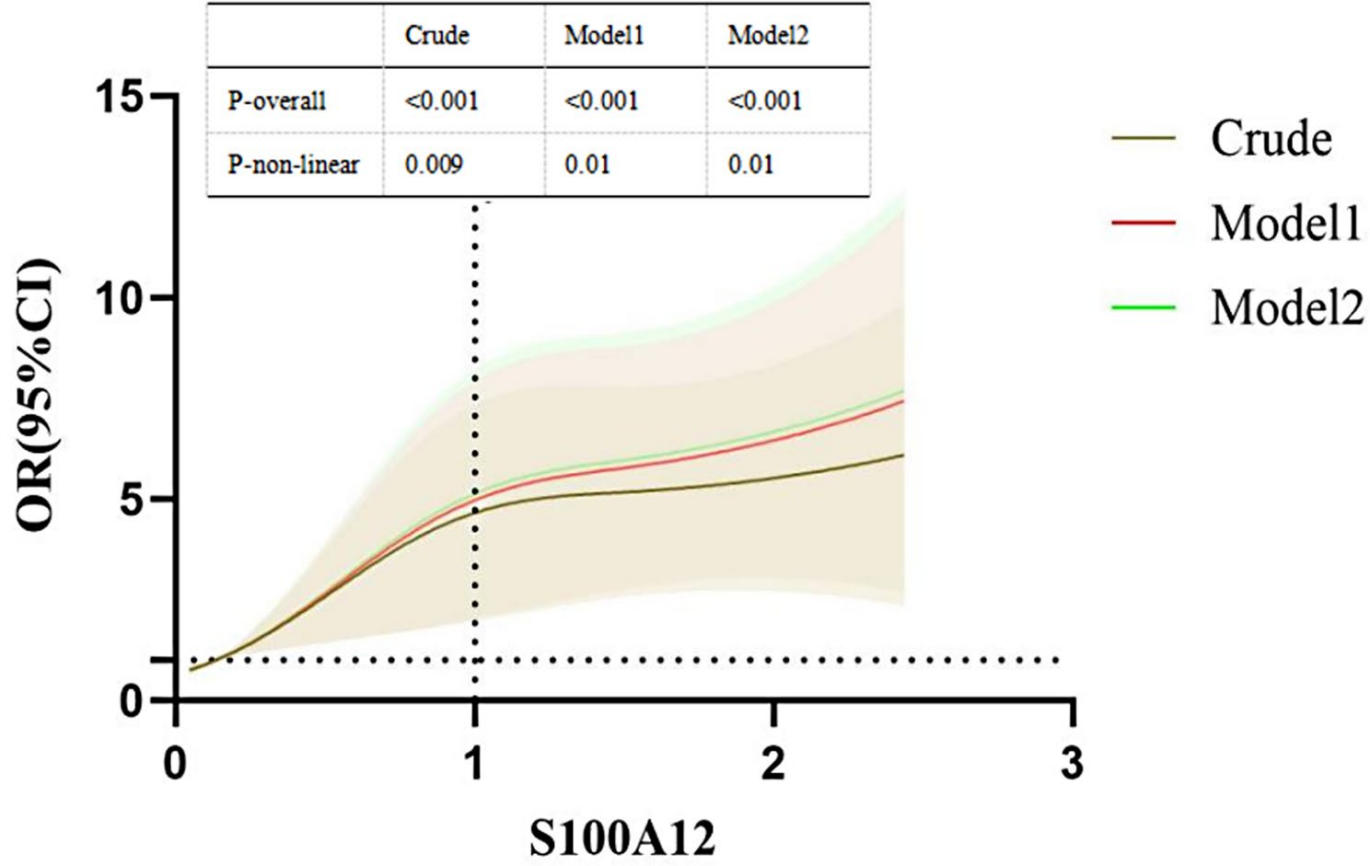
RCS plot showing the association between S100A12 levels and risk of PAD

As shown in Fig. 4, a nonlinear relationship between S100A12 level and risk of PAD was suggested by the RCS curves of the three models (all *p*-non-linear value < 0.05). The RCS curves showed that the positive correlation between S100A12 and the risk of PAD was stronger when the S100A12 level was less than 1.00ng/cL, whereas when S100A12 level was greater than 1.00ng/cL, the correlation between the two was weaker.

## Discussion

This study, for the first time, showed a positive correlation between S100A12 and PAD risk among patients with dyslipidemia. Further subgroup analyses showed that the positive association between S100A12 and the risk of PAD was significant in all subgroups, regardless of whether HDL-C levels were less than 1.03 mmol/L, age greater than 60 years, and presence of diabetes or hypertension. In addition, RCS curves suggested a better positive correlation between the two at S100A12 less than 1.00ng/cL and a weaker relationship at greater than 1.00ng/cL.

The relationship between S100A12 and coronary atherosclerosis has been mentioned in previous studies [22–24]. The study by Zhang et al. suggests that S100A12 can be elevated within 2 h after the onset of symptoms in ST-segment elevation myocardial infarction (STEMI), and that S100A12 can diagnose STEMI more quickly compared to other biomarkers [22]. Hu et al. found S100A12 to be a reliable predictor of early prognosis in STEMI patients by bioinformatic analysis [23]. It has also been shown that increased levels of S100A12 is an independent predictor of in-stent restenosis in patients who have received coronary drug-eluting stent implantation [24]. However, PAD, as an important risk factor for cardiovascular disease, has not received enough attention in previous studies, so compared with previous studies, the present study focused more on patients with PAD. The correlation between S100A12 and PAD has been previously reported in several diseases [16, 17, 25]. Previous studies have suggested that higher S100A12 levels in patients with diabetes accelerate the development of PAD in patients, ultimately advancing the event of amputation [16]. It has also been shown that in patients with end-stage renal disease, there is a positive correlation between S100A12 levels and the risk of PAD [17]. A biochemical study suggested that S100A12 was one of the important genes in the co-occurrence of Crohn’s disease and PAD, and that neutrophil infiltration-mediated inflammation and immune modulation were important pathological processes involved [25]. The results of the present study suggested a positive correlation between S100A12 and the risk of PAD, which was consistent with previous studies [16, 17]. However, patients with dyslipidemia, as a high-risk group for the PAD, have not been sufficiently emphasized in previous studies. The present study focused on the patients with dyslipidemia, suggesting that S100A12 may play a broader role in PAD. S100A12 is expressed in myeloid cells including neutrophils and monocytes [11]. In the process of atherosclerosis, the accumulation of lipids such as LDL-C has a pro-inflammatory role [10]. During the inflammation, S100A12 acts as a cytokine by binding to cell surface receptors such as RAGE and toll-like receptor-4 (TLR-4). It has been shown that S100A12 binding to RAGE promotes the secretion of inflammatory factors such as IL-6 [26, 27]. This pro-inflammatory process may play an important role in endothelial damage and atherosclerotic plaque progression [28]. Tomohiro Komatsu et al. showed that the use of atorvastatin significantly reduced circulating levels of S100A12 [29], which corroborates the findings of the present study.

Interestingly, the present study found that S100A12 was significantly elevated in dyslipidemia PAD patients, but S100A8/A9, also as calcium-binding proteins which was reported to act an important role in oxidative stress [30], was not significantly elevated in these patients (*p* value = 0.069). The speculation may be twofold: first, since the *p* value is close to 0.05, it is reasonable to speculate that there is, in fact, a correlation between S100A8/9 and the risk of PAD. Because the sample size was not large enough, there was no significant correlation between the two in the database of the present study and a significant correlation between the two may be found after further expansion of the sample. Secondly, it has been proposed that compared to LDL-C, S100A8/A9 is more sensitive to oxidation, and therefore S100A8/A9 aggregation in atherosclerotic plaques would contribute to oxidant scavenging, resulting in less oxidative stress damage received by tissue cells during inflammation [31]. In contrast, S100A12 is more resistant to oxidation, which may explain the significantly higher risk of PAD in those with higher levels of S100A12, rather than higher S100A8/A9 [32].

In addition, after univariate logistic regression analyses, it was also found that lower HDL-C levels and DM were independent risk factors for PAD. A number of previous studies have shown similar findings [33, 34]. Increased blood glucose in diabetic patients may contribute to the formation of PAD by advanced glycation end-products (AGE) generation, oxidative stress, and epigenetic changes [35]. HDL-C reverses peripheral vascular atherosclerosis through reverse cholesterol transport, anti-inflammatory, antioxidant, endothelial protection, and antithrombotic effects [36]. Decreasing HDL-C therefore increases the risk of PAD.

It is noteworthy that in the present study HA1c levels were generally high in the PAD and control groups [7.90% (7.20%,9.00%) in the PAD group and 7.50 (6.20,8.70) in the control group]. Additionally, although the level of HA1c was significantly higher in the PAD group compared to the control group, HA1c was found to have a non-significant effect on the risk of PAD by univariate logistic regression analysis (*p* value = 0.088), which was inconsistent with previous finding [6]. We analyzed that this was due to the fact that in this retrospective study, clinicians often tend to check HA1c in patients with DM or risk factors for DM, whereas other patients do not routinely have their HA1c checked. And for these missing data, available case analysis was taken in the present study. Since the missing data were not randomly distributed, so the missing not at random (MNAR) leads to increased bias. Despite the large MNAR-induced bias in HA1c, we thought that HA1c could still reflect the effect of glycemic control on the risk of developing PAD to some extent, and therefore we still performed a univariate regression analysis of HA1c in the study. However, since HA1c was not measured in patients with relatively normal blood glucose, the statistical validity of the effect of HA1c on the risk of PAD was underestimated, leading to negative results suggested by univariate logistic regression analysis and we did not include HbA1c as a predictor in the subsequent prediction model.

The results of the subgroup analyses suggested that S100A12 was positively associated with the risk of PAD in all subgroups grouped by HDL-C, hypertension, diabetes, and age. Notably, the correlation between S100A12 and the risk of PAD may be stronger in subgroups with lower HDL-C levels (*p* value = 0.094). It has been suggested that HDL-C not only acts as a cholesterol reverse transporter during atherosclerosis but also inhibits inflammatory pathways in several ways [37]. Firstly, HDL-C activates endothelial nitric oxide synthase (eNOS) to maintain endothelial cell integrity and reduce the inflammatory response triggered by endothelial cell dysfunction [38]. Secondly, HDL-C reduces inflammation by preventing ox-LDL-mediated expansion of granulocyte monocyte progenitors (GMPs) through multiple pathways [39, 40]. In addition, macrophages exhibit pro- and anti-inflammatory properties depending on the environment, while HDL-C alleviates the pro-inflammatory characteristics of macrophages through multiple pathways [41–43]. Since S100A12 is a pro-inflammatory factor secreted by granulocytes, it is reasonable to speculate that the inflammatory response during plaque progression is not affected by the various anti-inflammatory mechanisms mediated by HDL-C in populations with lower levels of HDL-C, thus leading to a stronger correlation between S100A12 and PAD risk.

Meanwhile, the RCS curves showed that the correlation between S100A12 and PAD risk was non-linear. Such a nonlinear relationship between the two was not found in previous studies. This suggested that changes in circulating levels of S100A12 did not have a constant but limited effect on the risk of PAD. When S100A12 is elevated above 1.00ng/cL, its effect on the risk of PAD becomes weak. Previous studies have shown a nonlinear correlation between the systemic inflammation response index (SIRI) and the occurrence of revascularization [44], suggesting that the inflammatory response plays a nonlinear role in the process of vascular endothelial injury, which is similar to the results of the present study. It is reasonable to speculate that as S100A12 levels rise during the development of PAD, its binding receptors such as TLR-4 and RAGE may become saturated, which leads to saturation of the inflammatory response it mediates. Thus the continued rise in S100A12 levels no longer has a significantly impact on the risk of PAD.

The present study has several strengths. Firstly, this study for the first time found a significant correlation between S100A12 and the risk of PAD among patients with dyslipidemia, confirming that S100A12 plays an important role in the atherosclerotic process of peripheral arteries induced by LDL-C. Secondly the results were more reliable by logistic regression after adjusting other covariates and by subgroup analysis. Finally this study used RCS to explore the non-linear relationship between S100A12 and PAD risk and find the cutoff of the change of correlation between the two, which made the results of this study more accurate and can better guide the assessment of PAD risk and subsequent studies.

Limitations in this study also need to be noted. Firstly, this study is retrospective and there will be incomplete data collection and recall bias, leading to compromised results. In addition when dealing with missing data this study used available case analysis, which further exacerbated the bias. Secondly, this study only collected single-center samples, thus leading to selection bias. Finally, many factors affect the risk of PAD, and although as many factors as possible were collected for inclusion in this study, there is still a range of factors that were not included in the study, which could also affect the final results.

Based on the results of this study, there are several areas for further research. Firstly, multicenter prospective studies are needed to further validate the results of this study. In addition further follow-up is necessary for exploring the impact of S100A12 levels on the prognosis of PAD patients. Finally, the present study revealed a nonlinear relationship between S100A12 and PAD risk, and further studies are needed to investigate the molecular biological mechanisms underlying this nonlinear relationship.

## Conclusion

In conclusion, elevated S100A12 level is an independent risk factor for PAD in patients with dyslipidemia. In different subgroups, S100A12 was significantly and positively associated with the risk of PAD after adjusting for different factors. There is a non-linear relationship between S100A12 and the risk of PAD, with a stronger positive correlation at S100A12 levels below 1.00ng/cL. These findings implied that S100A12 is a potential biomarker for identifying patients with dyslipidemia who are at high risk of developing PAD. They also implied that S100A12 levels can be routinely monitored in dyslipidemic populations for the early detection of PAD and to guide the management of PAD. Finally, the results of this study emphasize that inflammation in dyslipidemia patients plays an important role in the development of PAD, suggesting that lipid control and immunomodulation may be effective in the prevention of PAD.

## Electronic supplementary material

Below is the link to the electronic supplementary material.

Supplementary Material 1

## Data Availability

Data is provided within the manuscript.

## Abbreviations

ABI: Ankle-brachial blood pressure index
AGE: Advanced glycation end-products
AIS: Abbreviated injury scale
BMI: Body mass index
BUN: Blood urea nitrogen
DM: Diabetes mellitus
EDTA: Ethylenediaminetetraacetic acid
eNOS: Endothelial nitric oxide synthase
GMPs: Granulocyte monocyte progenitors
HA1c: Glycosylated hemoglobin
HDL-C: High-density lipoprotein cholesterol
HTN: Hypertension
ICAM-1: Intercellular adhesion molecule-1
IL-1β: Interleukin-1β
LDL-C: Low-density lipoprotein cholesterol
MAPK: Mitogen-activated protein kinase
MNAR: Missing not at random
NF-κB: Nuclear factor-κB
Nox1: NADPH oxidase 1
PAD: Peripheral arterial disease
RAGE: Receptor for advanced glycation end products
RCS: Restricted cubic spline
ROS: Reactive oxygen species
SIRI: Systemic inflammation response index
SOFA: Sequential organ failure assessment
STEMI: ST-segment elevation myocardial infarction
TC: Total cholesterol
TG: Triglyceride
TLR-4: Toll-like receptor-4
TNF-α: Tumor necrosis factor-α
VCAM-1: Vascular cell adhesion molecule-1
