# Supplementary material for: Association between S100A12 and risk of peripheral arterial disease in patients with dyslipidemia: a cross-sectional study
Source: BMC Cardiovasc Disord. 2025 Apr 24;25:313. doi: 10.1186/s12872-025-04752-2 (PMC12020102; doi:10.1186/s12872-025-04752-2)
Supplement: Supplementary file 1 — Supplementary Material 1 [file 12872_2025_4752_MOESM1_ESM.docx]

| **sTable1. Lipid Management Targets for Patients in Different Risk Stratifications** | |
| --- | --- |
| Risk Stratification | Lipid Management Targets |
| Low Risk | <3.4mmol/L |
| Moderate and High Risk | <2.6mmol/L |
| Very High Risk | <1.8mmol/L |
| Ultra-High Risk | <1.4mmol/L |

| **sTable2a. Risk Stratification for Patients Without a History of ASCVD (Primary Prevention Patients)** | | | | |
| --- | --- | --- | --- | --- |
| Risk Factors | TC <4.1 or LDL-C <2.6(mmol/L) | | TC 4.1–5.1 or LDL-C 2.6–3.3(mmol/L) | TC 5.2–7.1 or LDL-C 3.4–4.8(mmol/L) |
| No Hypertension |  | |  |  |
| 0–1 | Low Risk (<5%) | | Low Risk (<5%) | Low Risk (<5%) |
| 2 | Low Risk (<5%) | | Moderate Risk (5%–9%) | Moderate Risk (5%–9%) |
| ≥3 | Moderate Risk (5%–9%) | | High Risk (≥10%) | High Risk (≥10%) |
| Hypertension |  | |  |  |
| 0–1 | Low Risk (<5%) | | Moderate Risk (5%–9%) | Moderate Risk (5%–9%) |
| ≥2 | High Risk (≥10%) | | High Risk (≥10%) | High Risk (≥10%) |
|  | | | | |
| **sTable2b. Risk Stratification for Patients With a History of ASCVD (Secondary Prevention Patients)** | | | | |
|  | | ≥2 severe ASCVD events ​ | 1 severe ASCVD event + ≥2 high-risk factors | Other |
| Risk Stratification | | Ultra-High Risk | Ultra-High Risk | Very High Risk |
| **Definition:**  **Severe ASCVD Events:**   1. Recent ACS (<1 year) 2. Prior MI (excluding recent ACS) 3. Ischemic stroke history 4. Symptomatic PAD with revascularization/amputation.   **High-Risk Factors:**   1. LDL-C >1.8 mmol/L with recurrent ASCVD 2. Premature CHD (male <55, female <65) 3. Familial hypercholesterolemia or LDL-C ≥4.9 mmol/L   4.Prior CABG/PCI  5. CKD stage 3–4  6. Smoking | | | | |
